# CD68- and CD163-positive tumor infiltrating macrophages in non-metastatic breast cancer: a retrospective study and meta-analysis

**DOI:** 10.7150/jca.33914

**Published:** 2019-07-23

**Authors:** Chao Ni, Liu Yang, Qiuran Xu, Hongjun Yuan, Wei Wang, Wenjie Xia, Dihe Gong, Wei Zhang, Kun Yu

**Affiliations:** 1 Key Laboratory of Tumor Microenvironment and Immune Therapy of Zhejiang Province, Second affiliated hospital, Zhejiang University, Hangzhou, Zhejiang, 310009; 2 Key Laboratory of Tumor Molecular Diagnosis and Individualized Medicine of Zhejiang Province; 3 Department of Breast Surgery, Zhejiang Provincial People's Hospital, People's Hospital of Hangzhou Medical College, Hangzhou 310003, China; 4 Department of Pathology, Zhejiang Provincial People's Hospital, People's Hospital of Hangzhou Medical College, Hangzhou 310003, China; 5 Department of Thyroid and Breast Surgery, Affiliated Cixi Hospital, Wenzhou Medical University, Cixi Zhejiang 315300, China; 6 Department of Endocrinology, Zhejiang Provincial People's Hospital, People's Hospital of Hangzhou Medical College, Hangzhou 310003, China

**Keywords:** breast cancer, macrophage, CD68, CD163

## Abstract

Studies have indicated the significance of tumor associated macrophages (TAMs) in breast cancer; however, inconsistent results still exist. We retrospectively reviewed the macrophage distribution in 1579 breast cancer specimens with anti-CD68 or anti-CD163 immunohistochemical staining, and further analyzed the overall survival data. Furthermore, we performed a retrospective study and systematic review of the published studies on CD68- and CD163-positive macrophages in non-metastatic breast cancer. 13 studies with 5116 patients were included in this meta-analysis. Our own data revealed a high density of both CD68- and CD163-positive TAMs that was significantly related to lymph node metastasis (CD68, *P* = 0.003; CD163, *P* < 0.001); high Ki67 (CD68, *P* = 0.026; CD163, *P* < 0.001), poor histological grade (CD68, *P* < 0.001; CD163, *P* < 0.001) and hormonal receptor negativity (CD68, *P* < 0.001; CD163, *P* < 0.001); only CD163-positive TAMs were associated with poor overall survival (*P* = 0.003). Nonetheless, the meta-analysis only found that CD68- and CD163-positive TAMs were associated with high Ki67 [CD68, Relative risk (RR): 1.18, 95% confidence interval (CI): 1.09-1.28; CD163, RR: 1.75, 95% CI: 1.39-2.20], advanced histological grade (CD68, RR: 1.72, 95% CI: 1.46-2.03; CD163, RR: 1.99, 95% CI: 1.35-2.94) and low hormonal receptor levels (CD68, RR: 0.75, 95% CI: 0.69-0.82; CD163, RR: 0.82, 95% CI: 0.74-0.90), but not lymph node metastasis and HER2 expression. This meta-analysis further supports the clinical significance of TAMs in breast cancer, and both CD68- and CD163-positive TAMs could be prognostic markers in non-metastatic breast cancer.

## Introduction

Breast cancer has one of the highest incidences among malignant diseases for women worldwide. Despite the advancement of therapeutic strategies, according to the Surveillance, Epidemiology, and End Results (SEER) Program, the 5-year survival rate for metastatic breast cancer is still around 25% 1. Most of the cancer-related deaths are due to rapid progression and chemoresistance. Currently, continuous efforts are being made to illuminate the underlying molecular mechanisms of breast cancer.

One of the most promising aspects is the tumor microenvironment (TME). The TME is composed of various elements around tumor cells, including fibroblasts, adipocytes, blood vessels and immune cells 2. The importance of the immune microenvironment makes it a promising predictive marker and therapeutic target. For example, a subset analysis of the GeparQuattro trial found that each 10% increase in tumor infiltrating cells (TILs) was related to a higher pathological complete response (pCR) rate after neoadjuvant trastuzumab and chemotherapy 3. A higher number of FOXP3+ TILs was found to be associated with higher-risk clinicopathological factors and ER negativity 4. One study revealed that high expression of CXCL13 by Tfh cells in breast cancer was associated with a higher pCR rate to chemotherapy and better DFS 5. Among these immune cells in breast cancer, tumor-associated macrophages (TAMs) were reported to be the predominant component, comprising more than 50% of the cell mass in some cases 6.

In the tumor microenvironment, macrophages are converted into two subtypes: M1 and M2 macrophages 6. M1-like polarized macrophages promote the type-I immune response against bacterial, protozoal and viral infections and tumor cells, while the M2-like polarized macrophages have an anti-inflammatory function and regulate tissue remodeling, angiogenesis, and wound healing. In breast cancer, most of these TAMs are M2-like polarized macrophages and exert tumor-promoting functions. Although there have been dozens of publications on the clinicopathological and prognostic value of TAMs in breast cancer, most of them are limited in numbers. In addition, inconsistent TAM markers (CD68+/CD163+/CD206+) and some contradictory outcomes render use in clinical practice difficult. Here, we retrospectively studied the distribution of both CD68+ and CD163+ TAMs in 1579 early stage breast cancer specimens, which currently represents the largest sample size to date. Furthermore, we also performed a systematic review of published research to clarify the clinicopathological association and prognostic significance of CD68+ and CD163+ TAMs in early stage breast cancer.

## Materials and Methods

### Patient characteristics

A total of 1672 specimens of non-metastatic invasive breast cancer patients (from Jan 2007 to Feb 2013) from Zhejiang Provincial People's Hospital and Zhejiang Tiantai People's Hospital were retrospectively reviewed, of which 1579 patients with complete pathological features and 5-year follow-up were included in this study (Table 1). The immunohistochemistry outcome of 129 specimens was reviewed due to the lack of Her2 examination.

### Immunohistochemistry and macrophage quantification

The sections were obtained from paraffin-embedded tumor specimens and were stained by CD68 (P34810, Abgent, San Diego, CA) and CD163 (Q86VB7, Abgent, San Diego, CA) monoclonal antibodies. The samples were incubated first with antibodies and were then reacted with HRP-labeled secondary anti-mouse antibody (ASS1021, Abgent, San Diego, CA). The reaction was visualized using the DAB system. Normal mouse immunoglobulin (DAKO) was used as the negative control. The CD68- and CD163-positive macrophages were quantified in three randomized high-power fields (40 X) with two pathologists who were blinded to the clinicopathological features and prognosis of these patients.

### Search strategy

The literature search was performed by two reviewers independently through February 2018 with the MEDLINE, Ovid (including Embase 1974-2017) and Google Scholar databases as well as the China Knowledge Resource Integrated Database. The keywords applied were as follows: breast cancer, breast neoplasm and macrophage. These search terms were adapted to the proper syntax for each database. The included manuscripts were restricted to the English and Chinese languages, and the titles and abstracts of publications identified by the search were examined manually to exclude reviews, letters and articles on topics not relevant to this study.

### Eligibility criteria

All included studies in this meta-analysis met the following criteria: (1) focused on non-metastatic invasive breast cancer, (2) defined CD68 or CD163 expression in the tumor infiltrating macrophages, (3) clear analysis of correlations between CD68+ or CD163+ macrophages and clinicopathological features or survival outcomes (5-year overall or disease-free survival), and (4) published in English or Chinese.

To control for the quality of this meta-analysis, all of the enrolled studies were examined with seven key points provided by the Dutch Cochrane Centre: (1) a clear definition of study population and country of origin, (2) a clear definition of the type of carcinoma, (3) a clear definition of the study design, (4) a clear definition of the outcome assessment, (5) a clear definition of the cut-off of CD68 or CD163 expression on macrophages, (6) a clear definition of the method of CD68 or CD163 expression assessment, and (7) sufficient time for follow-up if survival data were presented.

### Data extraction

All data were extracted independently by two reviewers. The following data were extracted with a standardized method: author's name, publication year, country, and the number of total patients. In addition, in each group, T category (T1 and T2-4), N category (N0 and N1-3), Ki67, hormonal receptor status, HER2 expression, triple negative breast cancer, tumor grade (1-2 and 3), 5-year disease-free survival/recurrence-free survival and 5-year overall survival rate were evaluated. For studies that provided survival data with a Kaplan-Meier curve, GetData Graph Digitizer 2.24 software (http://getdata-graph-digitizer.com/) was used to digitize and extract the data from the Kaplan-Meier curves. We also tried our best to collect the missing information by emailing the corresponding authors.

### Quality assessment of primary studies

Two reviewers (Kun Yu and Chao Ni) independently evaluated the quality of the included studies using the Newcastle-Ottawa Quality Assessment Scale (NOS). Studies with NOS scores above 6 were identified as high-quality studies and disagreements were resolved by joint discussion.

### Statistical analysis

For the data provided by our group, the results are presented as the mean ± standard error, and the survival probabilities between the different groups were analyzed with the Kaplan-Meier method with GraphPad Prism 6.0. For the meta-analysis, the extracted data were analyzed with guidelines proposed by the Meta-Analysis of Observational Studies in Epidemiology group. Relative risk (RR) with a 95% confidence interval (95% CI) was determined with Review Manager 5.3. The heterogeneity among studies was measured by Q and I2 tests. A fixed or random model was used depending on the heterogeneity analysis. The potential for publication bias was also evaluated using the Begg rank correlation method and the Egger weighted regression method (Stata 11.0 software, Statacorp LLC, TX). Sensitivity analysis was conducted(Stata 11.0 software, Statacorp LLC, TX), in which individual studies were sequentially omitted. A P value <0.05 was considered statistically significant. All *P* values were two-tailed.

## Results

### Patients' characteristics

A total of 1579 specimens of non-metastatic invasive breast cancer patients (from Jan 2007 to Feb 2013) from Zhejiang Provincial People's Hospital and Zhejiang Tiantai People's Hospital were enrolled. The clinicopathological features of these cases are presented in Table 1. According to the guidelines of the National Comprehensive Cancer Network, 1503 patients received chemotherapy, while 23.4 percent of these patients received neoadjuvant therapy and the rest received adjuvant chemotherapy. A total of 1171 patients received hormonal treatment, and 343 patients received anti-Her2 therapy. To avoid the influence of neoadjuvant chemotherapy on macrophages, only pre-chemotherapy specimens were collected.

### CD68/CD163 immunostaining and clinicopathological association

According to the immunostaining results, CD68- and CD163-positive cells were all considered as TAMs and M2-like macrophages. Moreover, all macrophages were identified by morphological appearance. The medians of CD163-positive, CD68-positive and CD163/CD68 double-positive macrophages per high power field were 21 (3-54), 33 (7-68) and 21 (3-49), respectively. In the subgroup analysis, a high number of CD68-, CD163- or CD163/CD68 double-positive macrophages was defined as greater than the median, while a low number was defined as less than the median.

**Figure 1:**
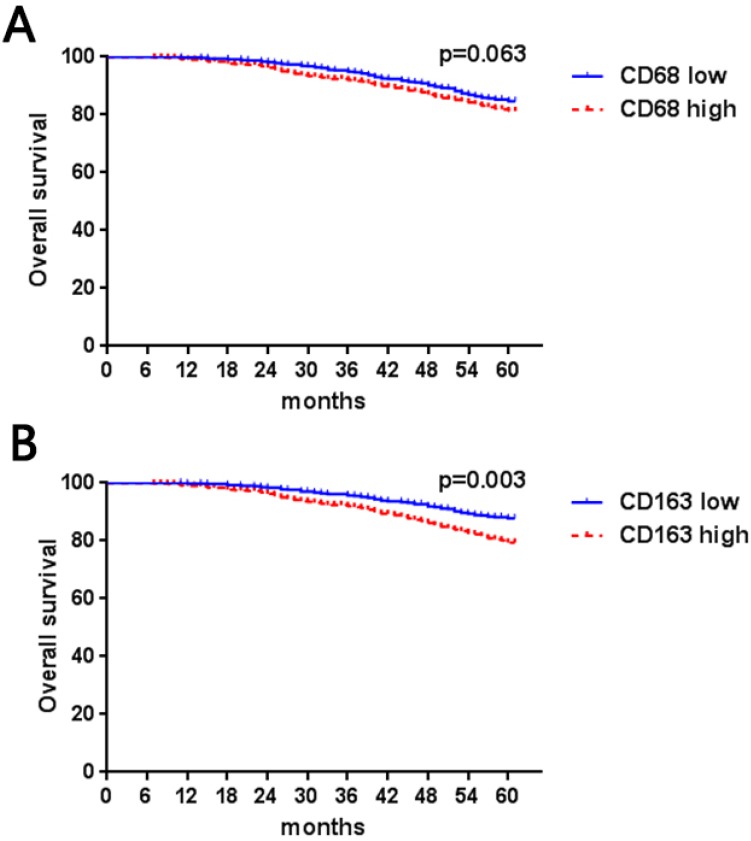
Kaplan-Meier Curve based on CD68- (a) or CD163- (b) positive macrophages in breast cancer

In our data (Table 1), high numbers of CD163- and CD68-positive TAMs were both significantly associated with lymph nodes metastasis (CD68, *P* = 0.003; CD163, *P* < 0.001), high Ki67 (CD68, *P* = 0.026; CD163, *P* < 0.001), poor histological grade (CD68, *P* < 0.001; CD163, *P* < 0.001) and hormonal receptor negativity (CD68, *P* < 0.001; CD163, *P* < 0.001), while a high number of CD163 TAMs was correlated with lymph node metastasis (CD68, *P* = 0.07; CD163, *P* = 0.022). CD68- and CD163-positive cells were not correlated with tumor size (CD68, *P* = 0.19; CD163, *P* = 0.27) or HER2 expression status (CD68, *P* = 0.33; CD163, *P* = 0.18). Furthermore, we evaluated the association between CD68- and CD163-positive TAMs. We found that CD68 and CD163 double-positive macrophages were more likely to be distributed in tumors with lymph node metastasis (*P* < 0.001) and had high histological grade (*P* < 0.001) and Ki67 index (*P* < 0.001) as well as negative HR expression (*P* < 0.001). Furthermore, our results indicated that high numbers of CD163-positive TAMs were associated with short 5-year overall survival (*P* = 0.003, Figure 1B); however, although there was a trend in which patients with a higher number of CD68-positive cells had poorer survival rates (*P* = 0.063), the result did not reach statistical significance (Figure 1A).

### Search results

**Figure 2:**
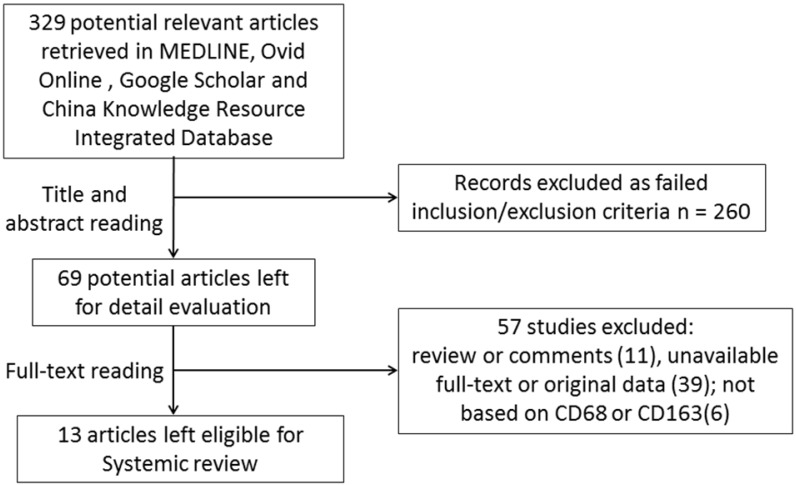
Flow chart for studies selection.

Here, we performed a comprehensive search of the published literature. Initially, 329 publications were retrieved by our primary research. Thereafter, 260 studies were excluded based on abstracts if they were basic research studies, reviews or written in a language other than English or Chinese. Then, the full-texts of the remaining 69 studies were carefully reviewed. A total of 57 studies were excluded because they did not provide a defined cut-off of macrophage levels, detailed information about clinicopathological features, the survival rate was unavailable, or macrophages were not detected using CD68 or CD163. Although we tried to contact all of the corresponding authors by e-mail to fill in the data table, we were unable to contact them within 8 weeks. Finally, including the current work, a total of 13 retrospective studies from 2005 to 2018 including 5116 non-metastatic breast cancer patients were enrolled in this meta-analysis (Figure 2). Seven studies comprising 2371 subjects were conducted in Asia, and 5 studies comprising 2745 subjects were conducted in Western countries (Europe or the USA). All studies used immunohistochemistry to detect CD68- or CD163-positive macrophages in tumor specimens. The cut-off value for CD68- and CD163-positive cell numbers varied among studies, and seven studies defined the cut-off value as the median number 7-14, while the other studies defined the optimal cut-off value with various statistical approaches 15-18.

### Correlation of TAMs and clinicopathological parameters

**Figure 3:**
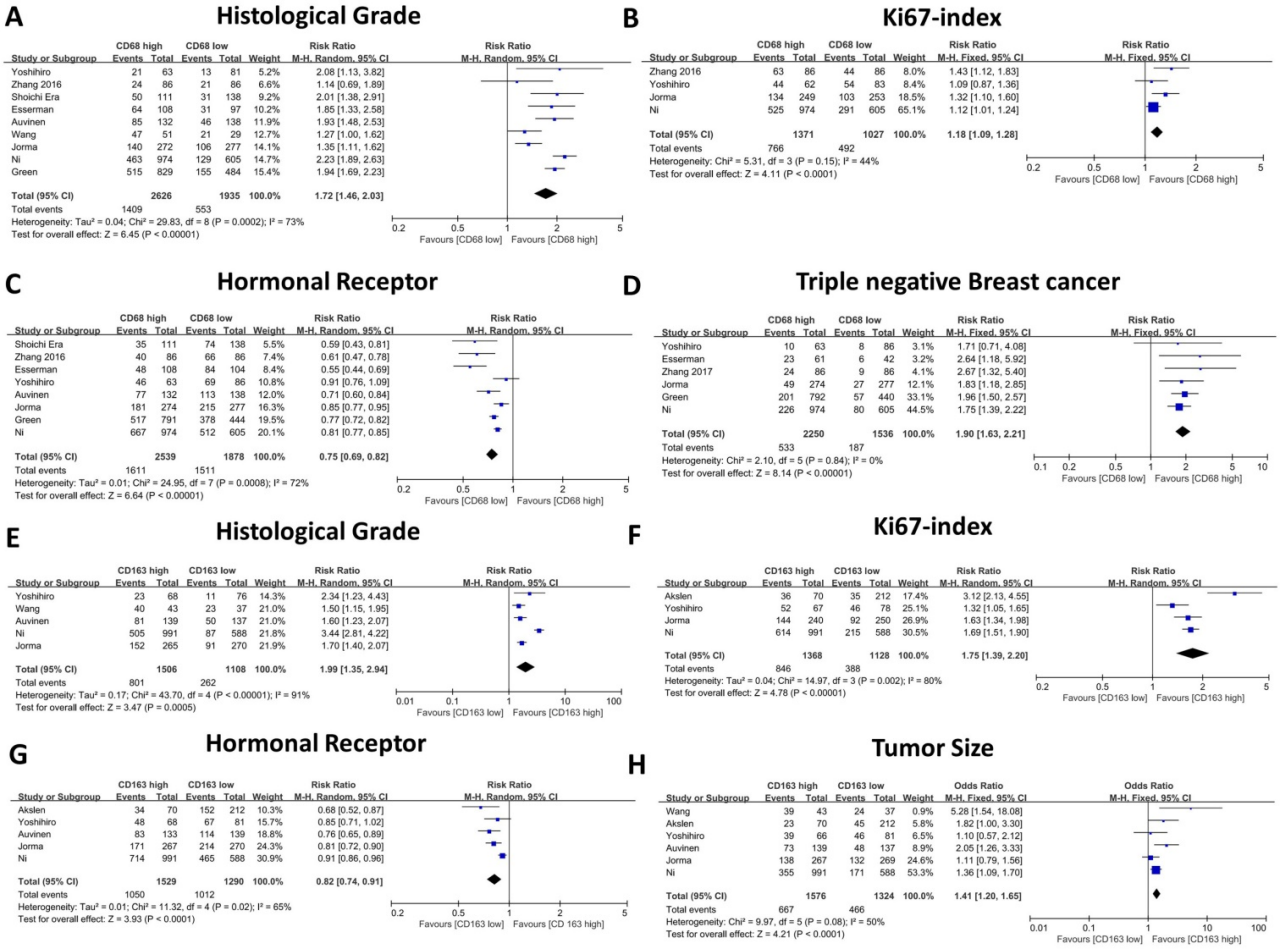
The forest plot of RRs was assessed for association between tumor infiltrating macrophages and clinicopathological features. CD68+ macrophages were illustrated as Histological grade (A), Ki67 index (B), Hormonal receptor level (C), Triple negative breast cancer (D). CD163+ macrophages were illustrated as Histological grade (E), Ki67 index (F), Hormonal receptor level (G), Tumor size (H). Each result was shown by the RR with 95% CIs (according to the fixed model or randomized model).

**Figure 4:**
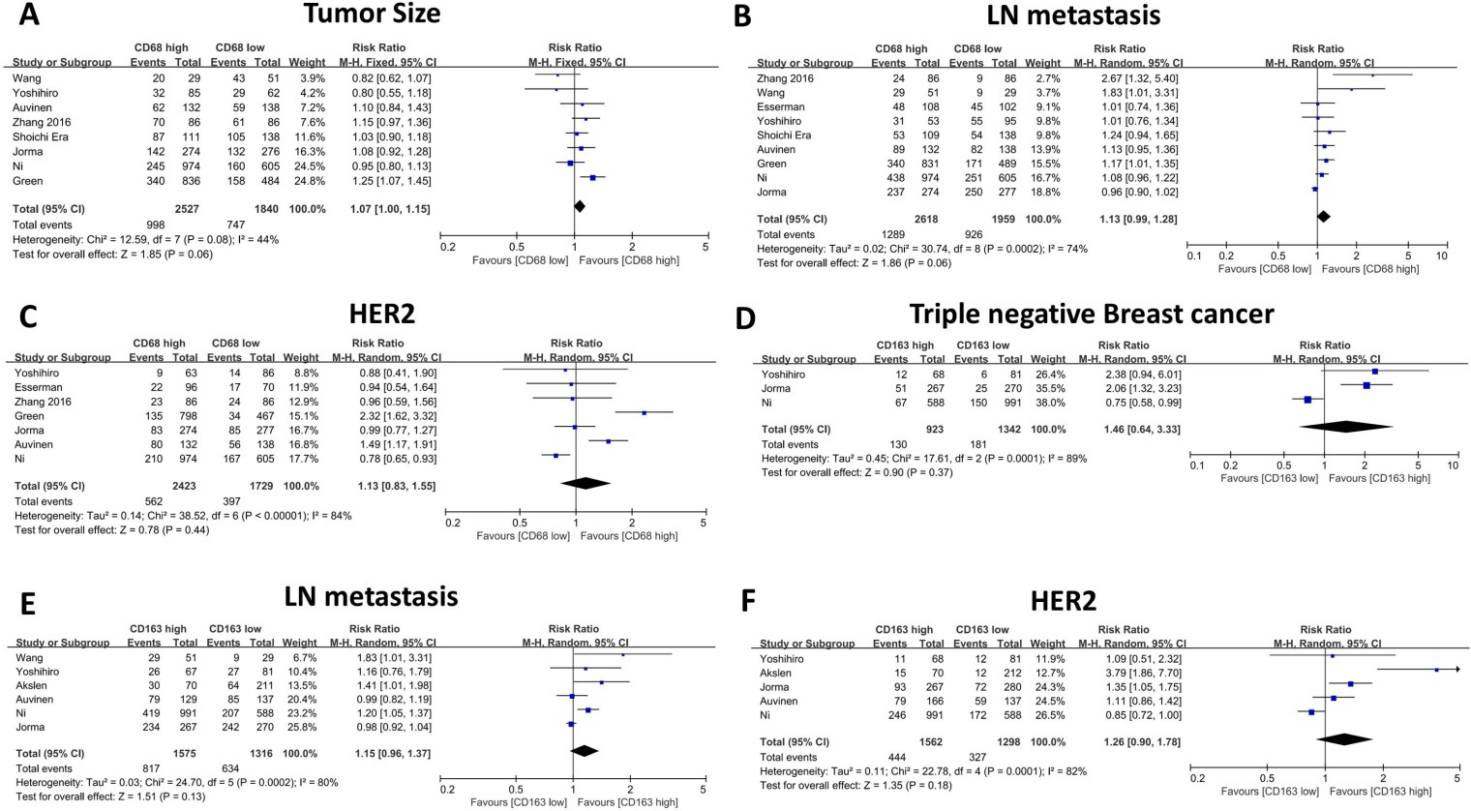
The forest plot of RRs was assessed for association between tumor infiltrating macrophages and clinicopathological features: CD68+ macrophages were illustrated as Tumor Size (A), Lymph node metastasis (B), Her2 expression (C); CD163+ macrophages were illustrated as Triple negative breast cancer (D).Lymph node metastasis (E), Her2 expression (F). Each result was shown by the RR with 95% CIs (according to the fixed model or randomized model).

The association between CD68- or CD163-positive TAMs and clinicopathological features, including T category, N category, histological grade, HR status, and HER2 and Ki67 expression, is illustrated in Table 2 and Table 3. First, we evaluated the correlation between CD68-positive macrophages and the above parameters, and the meta-analysis results revealed that high CD68+ macrophage infiltration indicated advanced histological grade (RR: 1.72, 95% CI: 1.46-2.03, Figure 3A), high Ki67 expression (RR: 1.18, 95% CI: 1.09-1.28, Figure 3B), negative HR expression (RR: 0.75, 95% CI: 0.69-0.82, Figure 3C) and high TNBC proportion (RR: 1.90, 95% CI: 1.63-2.21, Figure 3D). Meanwhile, CD68+ macrophages failed to show a significant relationship with T category (RR: 1.12, 95% CI: 1.00 -1.15, Figure 4A), axillary lymph node metastasis (RR: 1.13, 95% CI: 0.99-1.28, Figure 4B) and HER2 expression (RR: 1.13, 95% CI: 0.83-1.55, Figure 4C).

Thereafter, we evaluated the association between CD163-positive TAMs and the above clinicopathological parameters, and our results indicated similar results. High CD163-positive TAM infiltration correlated with advanced histological grade (RR: 1.99, 95% CI: 1.35-2.94, Figure 3E), high Ki67 expression (RR: 1.75, 95% CI: 1.39-2.20, Figure 3F) and negative HR expression (RR: 0.82, 95% CI: 0.74-0.90, Figure 3G); however, unlike CD68+ TAMs, CD163-positive TAMs were not found to be related to TNBC proportion (RR: 1.46, 95% CI: 0.64-3.33, Figure 4D), which could be due to the inclusion of only three studies and great heterogeneity (I-square = 89%, *P* = 0.0001). Moreover, CD163-positive TAMs also revealed a significant relationship with T category (RR: 1.41, 95% CI: 1.20 -1.65, Figure 4H) but were not related to axillary lymph node metastasis (RR: 1.15, 95% CI: 0.96-1.37, Figure 4e) and HER2 expression (RR: 1.26, 95% CI: 0.90-1.78, Figure 4F).

### Impact of TAMs on disease-free survival and overall survival

**Figure 5:**
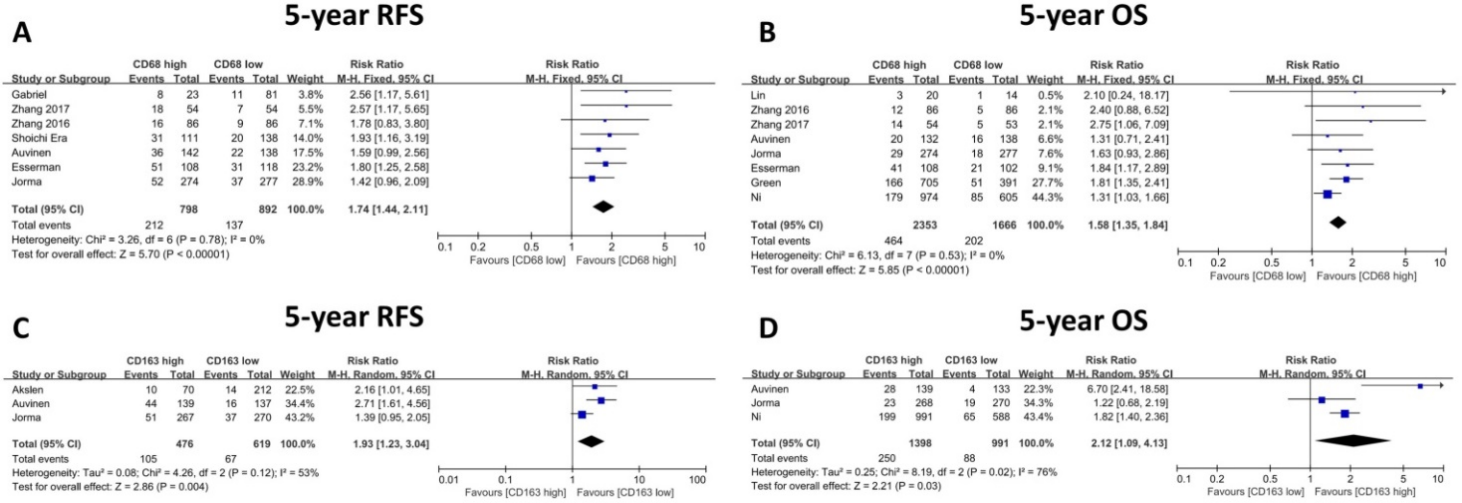
The forest plot of RRs was assessed for association between tumor infiltrating macrophages and survival rate: CD68+ macrophages were illustrated as 5-year Recurrence free survival (A), 5-year Overall survival (B); CD163+ macrophages were illustrated as 5-year Recurrence free survival (C), 5-year Overall survival (D).

**Figure 6:**
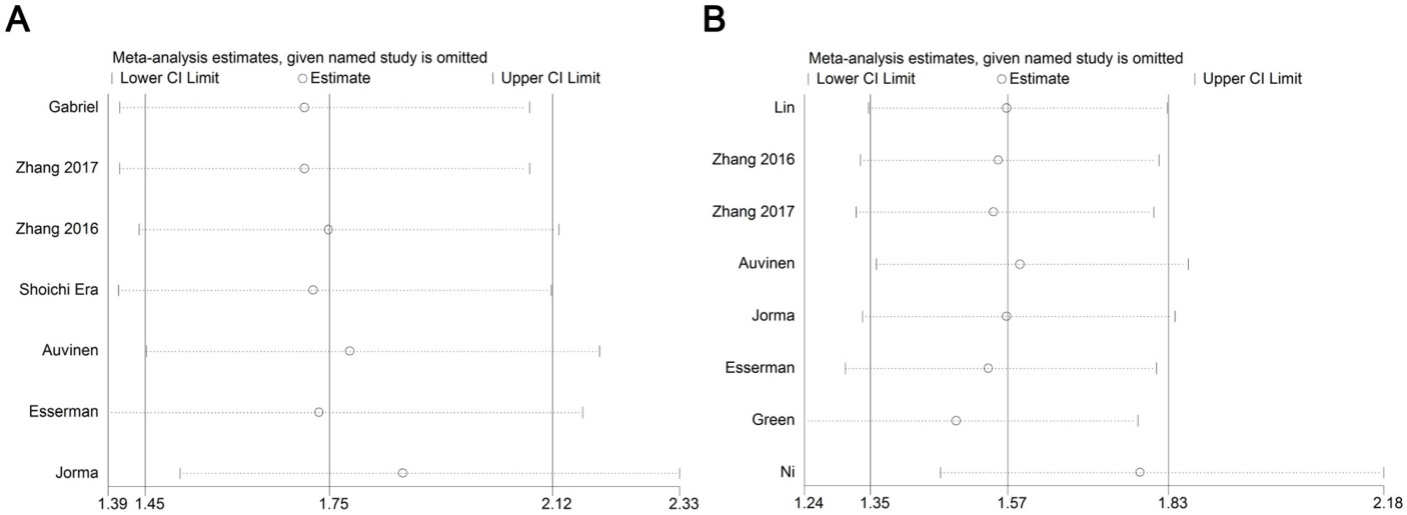
Forest plot of sensitivity analysis for influence of CD68+macrophage on RFS (A) and OS (B) with HR and 95%CI. HR >1 indicates better survival for the group.

Almost included publications presented the time to relapse event as RFS (recurrence free survival) 9, 10, 13-15, 17, while only one paper described it as DFS (disease-free survival) 18, according to the definition by NCI (National cancer Institute, https://www.cancer.gov/publications/dictionaries/cancer-terms/def/rfs), in most cases, RFS has the same meaning with DFS, herein we combined and analyzed the data described with RFS or DFS together. The relationship between 5-year relapse free survival (RFS) or overall survival (OS) and CD68-positive TAMs was presented in seven and eight studies, respectively. We found that the 5-year RFS rate was higher in patients with low CD68-positive TAM infiltration (CD68 low, 717/834, 86.0%; CD68 high, 614/815, 75.3%), which also indicated a significantly better outcome (RR: 1.74, 95% CI: 1.44-2.11, Figure 5A). Furthermore, the 5-year OS rates of the low and high CD68-positive TAMs groups were 87.9% (1466/1668) and 80.3% (1889/2353), respectively, and patients with high CD68-positive TAM infiltration showed poor prognosis (RR: 1.58, 95% CI: 1.35-1.84, Figure 5B). Sensitivity analysis was conducted here (Figure 6), and the result showed no clear variation in the combined HR and the result was robust. On the other hand, three studies gave the 5-year RFS and OS data based on CD163+ TAMs, and the results demonstrated that patients with low CD163-positive TAM density possess better 5-year RFS (CD163 low, 552/619, 89.2%; CD163 high, 371/476, 77.9%; RR: 1.93, 95% CI: 1.23-3.04, Figure 5C) and OS (CD163 low, 907/995, 91.2%; CD163 high, 1148/1398, 82.1%; RR: 2.12, 95% CI: 1.09-4.13, Figure 5D). Besides, since limited studies provided the data related to CD163+ TAMs, the sensitivity analysis was omitted.

## Discussion

Although the importance of the TME, especially TAMs in breast cancer, has been emphasized by extensive research, some controversies still exist regarding which biomarker could be applied for prognosis prediction as well as the relationship between these biomarkers and various breast cancer subtypes. In the present comprehensive meta-analysis, we report that both CD68- and CD163-positive macrophages are significantly associated with poor prognosis, advanced histological grade, high Ki67 expression and negative hormonal receptor expression in early stage breast cancer.

CD68 is recognized as a pan-macrophage marker and has been used to identify macrophages in routinely fixed paraffin-embedded tissue from various cancer types including breast cancer 16, 19. Since macrophages with different polarization statuses could exhibit opposite roles (“classically activated” M1 type with tumor-preventing or “alternatively activated” M2 type with tumor-promoting functions), compared with total macrophages, the M2 subtype of macrophages theoretically has a stronger correlation with advanced cancer stage and poor prognosis 10. Auvinen 9 reported that only CD163-positive macrophages were associated with tumor stage and nodal status in breast cancer. Moreover, although both CD68- and CD163-positive TAMs were related to short OS, the obsolete OS benefit at the end of the follow-up time was much lower in the CD163 macrophage group (CD163^high^ vs CD163^low^: 16%, CD68^high^ vs CD68^low^: 8%). Zhang et al. found that CD68-positive macrophages were only related to molecular subtypes of breast cancer and not with any other clinicopathological parameters 13. According to our meta-analysis results, we found that a high density of both CD68- and CD163-positive macrophages was significantly associated with high Ki67 expression, advanced histological stage, low HR expression and short OS, but not with tumor size, lymph node metastasis and HER2 expression.

The relationship between the pan-macrophage marker CD68 and some other pro-tumor macrophage markers, such as CD163, CD204 and CD206, has been studied previously. However, in most cases, the number of M2-type macrophages was lower than that of CD68-positive macrophages, although the densities of CD163 or CD204 macrophages could oftentimes be higher compared to CD68 macrophages 7, 20, 21. One explanation is that CD68 is a lysosomal-associated membrane protein and not a cell membrane protein^22^, and thus, it may be downregulated in some macrophages. In addition, some studies also evaluated the correlation between the ratio of CD163- to CD68-positive macrophages and clinicopathological features in malignant tumors. Zhang et al. 13 demonstrated that the density of CD206 and CD68 double-positive macrophages was significantly associated with high tumor histological grade, the Ki67 index and low hormonal receptor expression in breast cancer. In ER-negative subtypes, almost 70% of the macrophages stained positive for CD206. Takeya et al. 21 reported that CD68-positive TAMs also expressed a high level of CD163 in high grade gliomas, and this ratio was decreased in low grade tumors.

In our own data, we also found that the ratio of CD163- to CD68-positive macrophages was higher in high histological and Ki67 index breast cancer, where the proportion was nearly 80% in the HR-negative subtype and only about 40% in the HR-positive subtype (data not shown). In line with the high correspondence between these two markers in breast cancer, especially within the aggressive subtypes, our meta-analysis results revealed a similar correlation between CD68- and CD163-positive macrophages with clinicopathological parameters and prognosis.

Consistent with the previous report, our comprehensive analysis found that basal-like breast cancer cells showed a strong association with TAMs, especially CD163-positive macrophages. Compared with luminal-like breast cancer cells, basal-like breast cancer cells are more likely to express a broader range of receptors for macrophage-derived cytokines, which could recruit macrophages into the microenvironment 10, 23 and promote monocyte differentiation into M2-like macrophages 24. For example, it has been reported that S100A4, a small Ca2+-binding protein secreted by various cells into the tumor microenvironment, could activate breast cancer cells, especially the TNBC type, to promote macrophage conversion into the M2 type 25. TNBC could also cause down-regulation of citrulline metabolism and differentiation into M2-like macrophages with increased levels of various cytokines, such as IL-8, IL-6, CXCL10, CCL2 and CCL5 25, 26. On the other hand, the association between HER2 and TAMs remains undetermined among studies. Several studies reported that HER2+ breast cancer has more TAM infiltration (CD68-positive 10, 16; CD163-positive 9, 14, 16), while the majority of the included studies failed to reveal a significant relationship between HER2 expression and TAMs in breast cancer. In a mouse model of HER2+ breast cancer, researchers found that overexpression of HER2 could induce the production of CCL2 by cancer cells and that myeloid cells attract CD206+/Tie2+ macrophages that promote the epithelial to mesenchymal transition in HER2+ breast cancer cells 27. This report raised the hypothesis that HER2+ breast cancer cells attract and educate macrophages into CD206-positive but not CD163-positive M2-like cells, which was in line with the results from two clinical studies 13, 28 that found CD206-positive macrophages were more likely to be associated with HER2-positive breast cancer.

## Conclusions

In conclusion, the results of this meta-analysis further support the prognostic value of TAMs in breast cancer. However, this meta-analysis is subject to a few limitations. First, this meta-analysis is based on published literature, and thus, the individual patient data were unattainable, which could decrease the accuracy of the results. Second, about half of the included patients were from Asia, which may introduce bias. In spite of these limitations, our meta-analysis revealed that high infiltration of CD68- and CD163-positive TAMs was significantly related to advanced histological grade, a high Ki67 index, low HR expression, and adverse RFS and OS. Large interventional and prospective studies based on different subtypes of TAMs should be performed in the future to generate more accurate results.

## Figures and Tables

**Table 1:** Correlations between TAMs and clinicopathological parameters

	CD68 high	CD68 low	*P*-value	CD163 high	CD163 low	*P*-value	CD163/CD68		*P*-value
							high	low	
**Age(years)**									
**Median**	58	56		58	56		57	57	
**Tumor stage, no.(%)**									
**T1**	729	445	0.19	757	417	0.27	732	442	0.27
**T2**	145	88		149	84		157	76	
**T3**	76	49		70	55		64	61	
**T4**	24	23		19	28		19	28	
**Nodal status, no.(%)**									
**pN0**	526	364	0.003	509	381	<0.001	489	401	<0.001
**pN1-3**	458	231		482	207		480	209	
**Ki67, no.(%)**									
**≥14%**	525	291	0.026	614	202	<0.001	597	219	<0.001
**<14%**	449	314		377	386		386	377	
**Histological grade, no.(%)**									
**1-2**	511	476	<0.001	486	501	<0.001	469	518	<0.001
**3**	463	129		505	87		502	90	
**ER/PR status, no.(%)**									
**Positive**	667	512	<0.001	634	545	<0.001	658	521	<0.001
**Negative**	307	93		357	43		345	55	
**HER2 status, no.(%)**									
**Positive**	231	146	0.33	226	151	0.18	213	164	0.08
**Negative**	769	430		765	434		739	460	

**Table 2:** Main characteristics of included studies based on CD68 positive macrophages.

Author	Country	No.	Cut off (low/high)	T grade (1, >1)	Ki67 (high/low)	HR (pos/neg)	Her2+ (pos/neg)	TNBC/others	LN met. pos/neg	Grade (1,2/3)	5 year RFS (pos/neg)	5 year OS (dead/alive)	NOS
Komohara	Japan	149	Median (86,63)	L(33,29); H(53,32)	L(54,29); H(44,18)	L(69,17); H(46,17)	L(14,72); H(9,54)	L(8,78); H(10,53)	L(55,40); H(31,22)	L(68,13); H(42,21)	NA	NA	6
Lin*	China	34	median (14,20)	NA	NA	NA	NA	NA	NA	NA	NA	L(1,13); H(3,17)	6
Auvinen	Finland	276	median (138,132)	L(79,59); H(70,62)	NA	L(113,25); H(77,55)	L(56,82); H(80,52)	NA	L(82,56); H(89,43)	L(92,46); H(47,85)	L(22,116); H(36,96)	L(16,122); H(20,112)	7
Jorma	Finland	551	median (277,274)	L(144,132); H(132,142)	L(104,149); H(134,116)	L(215,62); H(181,93)	L(85,192);H(83,191)	L(27,250); H(49,225)	L(250,27); H(237,37)	L(171,106);H(132,140)	L(37,240); H(52,222)	L(18,259); H(29,245)	5
Green	UK	1322	Median (486,836)	L(326,158); H(489,340)	NA	L(378,66); H(517,274)	L(34,433); H(135,663)	L(57,385); H(201,591)	L(171,312);H(340,491)	L(329,155);H(314,515)	NA	L(51,340); H(166,539)	8
Gabriel	France	104	40% (81,23)	NA	NA	NA	NA	NA	NA	NA	L(11,70); H(8,15)	NA	6
Esserman	USA	210	Median (102,108)	NA	NA	L(84,20); H(48,60)	L(17,53); H(22,74)	L(6,36); H(23,38)	L(45,57); H(48,60)	L(66,31); H(44,64)	L(31,87); H(51,57)	L(21,81); H(41,67)	8
Era	Japan	249	Median (138, 111)	L(33,105); H(24,87)	NA	L(74,64); H(35,76)	NA	NA	L(54,84); H(53,56)	L(107,31); H(61,50)	H(31,80); L(20,118)	NA	5
zhang	China	172	Median (86,86)	L(25,61); H(16,70)	L(44,42); H(63,23)	L(66,20); H(40,46)	L(24,62); H(23,63)	L(9,77); H(24,62)	L(51,35); H(42,44)	L(65,21); H(62,24)	L(9,77); H(16,70)	L(5,81); H(12,74)	5
Wang	China	80	Median (29,51)	L(8,43); H(9,20)	NA	NA	NA	NA	L(9,20); H(29,22)	L(5,24); H(4,47)	NA	NA	6
Zhang*	China	108	Median (54,54)	NA	NA	NA	NA	NA	NA	NA	L(7,47); H(18,36)	L(5,49); H(14,40)	6
Ni	China	1579	Median (605,974)	L(445,160); H(729,245)	L(291,314); H(525,449)	L(512,93); H(667,307)	L(167,435);H(210,764)	L(80,525); H(226,748)	L(251,354);H(438,536)	L(476,129);H(511,463)	NA	L(85,520); H(179,795)	7

*, all included patients were TNBC; HR, hormonal receptor; LN met., lymph node metastasis; RFS, recurrent free survival; OS, overall survival; NA, not available.

**Table 3:** Main characteristics of included studies based on CD163 positive macrophages.

Author	Country	No.	Cut off (low/high)	T grade (1, >1)	Ki67 (high/low)	HR pos/neg	Her2+ pos/neg	TNBC/ others	LN met. pos/neg	Grade (1,2/3)	5 years RFS pos/neg	5 years OS dead/alive	NOS
Akslen	Norway	282	Median (212,70)	L(167,45); H(47,23)	L(35,177); H(36,34)	L(152,60);H(34,36)	L(12,200); H(15,55)	NA	L(64,147); H(30,40)	NA	L(14,198); H(10,60)	NA	8
Komohara	Japan	149	Median (81,68)	L(35,46); H(27,39)	L(46,32); H(52,15)	L(67,14);H(48,20)	L(12,69); H(11,57)	L(6,75); H(12,56)	L(27,54); H(26,41)	L(65,11); H(45,23)	NA	NA	6
Auvinen	Finland	276	Median (137,139)	L(89,48); H(66,73)	NA	L(114,25);H(83,50)	L(59,78); H(79,60)	NA	L(85,52); H(79,50)	L(87,50); H(58,81)	L(16,121); H(44,95)	L(4,133); H(28,111)	7
Jorma	Finland	537	Median (270,267)	L(137,132);H(129,138)	L(92,158); H(144,96)	L(214,56);H(171,96)	L(72,198); H(93,174)	L(25,245); H(51,216)	L(242,28); H(234,33)	L(179,91); H(113,152)	L(37,233); H(51,216)	L(19,251); H(23,245)	5
Wang	China	80	Median (37,43)	L(13,24); H(4,39)	NA	NA	NA	NA	L(9,20); H(29,22)	L(14,23); H(3,40)	NA	NA	6
Ni	China	1579	Median (588,991)	L(417,171);H(757,234)	L(202,386);H(614,377)	L(545,43); H(634,357)	L(172,416);H(246,745)	L(67,521); H(150,841)	L(381,207);H(509,482)	L(501,87); H(486,505)	NA	L(65,523); H(199,792)	7

HR, hormonal receptor; LN met., lymph node metastasis; RFS, recurrent free survival; OS, overall survival; TNBC, triple negative breast cancer; NA, not available.
